# Supplementary material for: A Critical View of the Application of the APEX Software (Aqueous Photochemistry of Environmentally-Occurring Xenobiotics) to Predict Photoreaction Kinetics in Surface Freshwaters
Source: Molecules. 2019 Dec 18;25(1):9. doi: 10.3390/molecules25010009 (PMC7017383; doi:10.3390/molecules25010009)
Supplement: Supplementary file 1 [file molecules-25-00009-s001.zip › 3.2.4_gcc-4.4.0.zip!/3.2.4_gcc-4.4.0/3.2.4_gcc-4.4.0/doc/octave/html/interpreter/Scatter-group.html]

Scatter group - Untitled


Next: Stair group,
Previous: Quiver group,
Up: Object Groups


---

##### 15.2.8.8 Scatter group

Scatter series objects are created by the `scatter` or `scatter3`
functions. A single hggroup element contains as many children as there are
points in the scatter plot, with each child representing one of the points.
The properties of the stem series are

`linewidth`: The line width of the line objects of the points. See Line Styles. `marker` `markeredgecolor` `markerfacecolor`: The line and fill color of the markers of the points. See Colors. `xdata` `ydata` `zdata`: The original x, y and z data of the stems. `cdata`: The color data for the points of the plot. Each point can have a separate color, or a unique color can be specified. `sizedata`: The size data for the points of the plot. Each point can its own size or a unique size can be specified. `xdatasource` `ydatasource` `zdatasource` `cdatasource` `sizedatasource`: Data source variables.
